# Heterogeneous Uptake of Nanoparticles in Mouse Models of Pediatric High-Risk Neuroblastoma

**DOI:** 10.1371/journal.pone.0165877

**Published:** 2016-11-18

**Authors:** Ketan B. Ghaghada, Zbigniew A. Starosolski, Anna Lakoma, Caterina Kaffes, Saurabh Agarwal, Khannan K. Athreya, Jason Shohet, Eugene Kim, Ananth Annapragada

**Affiliations:** 1 Department of Pediatric Radiology, Texas Children’s Hospital, Houston, Texas, United States of America; 2 Michael E. DeBakey, Department of Surgery, Division of Pediatric Surgery, Baylor College of Medicine, Houston, Texas, United States of America; 3 Department of Pediatrics, Baylor College of Medicine, Houston, Texas, United States of America; 4 Department of Pediatrics, Section of Hematology-Oncology and Center for Cell and Gene Therapy, Baylor College of Medicine, Houston, Texas, United States of America; 5 Texas Children's Cancer Center, Texas Children’s Hospital, Houston, Texas, United States of America; 6 University of Texas Medical School at Houston, The University of Texas Health Sciences Center at Houston, Houston, Texas, United States of America; University of Nebraska Medical Center, UNITED STATES

## Abstract

Liposomal chemotherapeutics are exemplified by DOXIL® are commonly used in adult cancers. While these agents exhibit improved safety profile compared to their free drug counterparts, their treatment response rates have been ~ 20%, often attributed to the heterogeneous intratumoral uptake and distribution of liposomal nanoparticles. Non-invasive and quantitative monitoring of the uptake and distribution of liposomal nanoparticles in solid tumors could allow for patient stratification and personalized cancer nanomedicine. In this study, the variability of liposomal nanoparticle intratumoral distribution and uptake in orthotopic models of pediatric neuroblastoma was investigated using a liposomal nanoprobe visualized by high-resolution computed tomography (CT). Two human neuroblastoma cell lines (NGP: a *MYCN*-amplified line, and SH-SY5Y a *MYCN* non-amplified line) were implanted in the renal capsule of nude mice to establish the model. Intratumoral nanoparticle uptake was measured at tumor ages 1, 2, 3 and 4 weeks post implantation. The locations of uptake within the tumor were mapped in the 3-dimensional reconstructed images. Total uptake was measured by integration of the x-ray absorption signal over the intratumoral uptake locations. Both tumor models showed significant variation in nanoparticle uptake as the tumors aged. Observation of the uptake patterns suggested that the nanoparticle uptake was dominated by vascular leak at the surface/periphery of the tumor, and localized, heterogeneous vascular leak in the interior of the tumor. Slow growing SH-SY5Y tumors demonstrated uptake that correlated directly with the tumor volume. Faster growing NGP tumor uptake did not correlate with any tumor geometric parameters, including tumor volume, tumor surface area, and R30 and R50, measures of uptake localized to the interior of the tumor. However, uptake for both SH-SY5Y and NGP tumors correlated almost perfectly with the leak volume, as measured by CT. These results suggest that the uptake of nanoparticles is heterogeneous and not governed by tumor geometry. An imaging nanoprobe remains the best measure of nanoparticle uptake in these tumor models.

## Introduction

Nanoparticle based therapies continue to be the focus of numerous drug development efforts. Twenty years after the approval of Doxil® (Stealth Liposomal Doxorubicin) for the treatment of Kaposi’s Sarcoma, a recent search of clinical trials.gov revealed that there were over 450 open clinical trials for liposomal drugs, including liposomal formulations of doxorubicin, paclitaxel, vincristine, bupivacaine, mitoxantrone, prednisolone, cytarabine, cortisol, irinotecan, amphotericin B, daunorubicin, amikacin, prostoglandin E1, and numerous siRNA’s. A meta-analysis of the last 30 years of data on nanoparticle delivery to tumors [1] shows that a very small fraction (<1%) of the injected dose actually deposits in the target tumor, yet interest in nanoparticle based therapies remains extremely high: even at this low deposited dose, the therapeutic index achievable with nanoparticle delivery promises to be higher than that from other delivery approaches, and a persuasive case for a 30 year research plan to increase the deposited dose has been presented [1]. A continuing problem however, is the variability of nanoparticle delivery, even within a given tumor type [2–4]. Both Abraxane and Doxil have a ~20% response rate, and it is yet unknown how much of this is due to low intra-tumoral delivery of the agent. A biomarker that predicts nanoparticle deposition in tumors could therefore be extremely useful, guiding therapy by determining which tumors are candidates for nanoparticle based therapy, by virtue of their receiving a larger fraction of the delivered dose of a nanoparticle based therapeutic. In 2009 we introduced the term “physiomarker” to describe such a parameter [5], distinguishing it from the typical biomarker that is usually characterized as a molecular or species concentration, predictive of pathology.

In previous work, we showed that a liposome encapsulating iodinated contrast agent, visualized by planar X-ray imaging, exhibited highly variable uptake in a MAT-B-III rat model of breast cancer [6–8], and that the uptake pattern was highly heterogeneous [9]. We also showed that when treated with liposomal doxorubicin, these individual tumors showed highly variable, but predictable response: the tumors that had the highest uptake of the iodinated liposome also had the best response to the liposomal doxorubicin, while those that showed low uptake of the iodinated particle exhibited poor response to the liposomal doxorubicin. We further showed that the uptake levels of nanoprobe were directly related to the level of angiogenesis as measured by the levels of VEGF-A, VEGF-B and VEGF-R1. MAT-B-III however, is a very rapidly growing tumor, with volumetric doubling in as few as 4–5 days, raising the question of whether such rapid growth resulted in uncontrolled angiogenesis, and perhaps more variability in leak through the fenestrations in rampant angiogenic vessels. In other previous work [10,11], we showed that non-orthotopic implanted tumors develop highly unusual vascular structures by co-option of surrounding and distant vessels, and by angiogenesis external to the tumor margin, thereby posing the question of whether tumors in their normally occurring environment also develop similar uptake heterogeneity. In a recent study, we showed that uptake heterogeneity also occurs in companion dogs with spontaneous tumors. However, to date, no studies of uptake heterogeneity have been conducted in pediatric solid tumors[12]. We hypothesize that a nanoparticle-based contrast agent enables non-invasive imaging and quantitative analysis of uptake and intra-tumoral distribution of nanoparticles. We therefore chose to test the variability of nanoparticle uptake in two orthotopic mouse models of high-risk neuroblastoma, the most common extracranial solid tumor in pediatric patients. We chose the SH-SY5Y cell line as an example of a *MYCN* non-amplified tumor with relatively slow growth, and the NGP cell line as an example of a *MYCN* amplified tumor with relatively fast growth, although not as fast as the MAT-B-III model we studied previously. We probed the uptake of liposomal nanoparticles in these tumors using a nanoprobe developed previously [6–9], but unlike previous work, we imaged the localization of the nanoparticles using computed tomography (CT), thus recording the locations of particle deposition in 3D, and enabling the quantification of total deposition by integration of the iodine signal. The radial distribution of particle deposition was measured by decomposing the tumor volume into outer-surface contoured concentric shells. We demonstrated that rapidly growing NGP tumors showed far higher variability in nanoparticle deposition than slower growing SH-SY5Y tumors. None of the geometric parameters of the tumors (diameter, surface area, volume, radial distribution of deposition) was successful in predicting the overall deposition. However, the volume of leak from the angiogenic vessels, as estimated by imaging, was able to accurately predict the overall deposition in both NGP and SH-SY5Y models, suggesting that an imaging based biomarker of nanoparticle deposition faithfully predicts the deposition of nanoparticle payload in the tumor.

## Materials and Methods

All animal studies were approved by the Institutional Animal Care and Use Committee (IACUC) of Baylor College of Medicine. The studies were in compliance with NC3RS-ARRIVE guidelines. Human neuroblastoma cells lines NGP and SH-SY5Y were used for tumor implantation. Xenograft studies were performed in four- to six-week-old female non-obese diabetic/severe combined immunodeficient (NOD/SCID) mice (NCI-Frederick, Frederick, MD). A total of 60 mice were used for the study. Tumor implantation was performed following a previously described orthotopic kidney capsule model of neuroblastoma [13,14]. Briefly, animals were pre-anesthetized with a cocktail of ketamine and xylazine, administered intra-peritoneally, at a dose of 80 mg/kg and 16 mg/kg, respectively. The animals were then brought to the operating area and placed under 1–1.5% isoflurane inhaled gas anesthesia delivered through a nose-cone setup for the surgical procedure. The kidney was externalized through a small lateral incision and one million NB cells (stably transduced with Luciferase), suspended in phosphate-buffered saline, were surgically injected in the sub-renal capsule. The kidney was replaced, skin and muscle were then closed with suture and the mice were allowed to recover. The surgical procedure is complete in under 2 minutes from the induction of anesthesia. Animals were covered in warm dry gauze with heating lamp and then placed in their cages to recover and awaken immediately after the procedure. Mice were monitored every 15 minutes for hypothermia and until they were awake. Animals were monitored daily until surgical wounds were healed and sutures removed. To minimize pain and distress, mice received coverage with buprenorphine (0.5 mg/kg sub-cutaneously every 12 hours) for a minimum of 48 hours after the procedures and then as necessary to control pain. Tumor-bearing mice were monitored three times a week until a palpable tumor nodule was present and then daily thereafter. Early euthanasia was considered if the mice were observed in moribund state, exhibited poor respirations, cachexia or obvious distress/pain, immobility, huddled posture, inability to eat, self-mutilation or body weight loss of more than 20%.

### Nanoparticle Contrast Agent

The nanoparticle contrast agent was prepared as per procedures described previously [10]. Briefly, 1,2-dipalmitoyl-sn-glycero-3-phospho- choline (DPPC), cholesterol, and 1,2-distearoyl-sn-glycero-3-phosphoethanolamine-N-[methoxy (polyethylene glycol)-2000] (DSPE- MPEG2000) were dissolved in ethanol at a molar ratio of 56:40:4. The ethanolic lipid solution was hydrated with an aqueous solution of iodixanol (550 mg I/mL) and then sequentially extruded at ~65°C on a Lipex Thermoline extruder (Northern Lipids, Vancouver, British Columbia, Canada) to size the liposomes to ~140 nm. The extruded liposomal nanoparticle solution was diafiltered against 150 mM saline solution to remove free iodixanol. The size distribution of liposomes in the final formulation was determined by dynamic light scattering (DLS). The iodixanol concentration in the final solution was determined by measuring UV absorbance at a wavelength of 245 nm. The average liposome size in the final formulation was 135±20 nm. The overall iodine concentration was 110 mg I/mL and the ratio of iodine to total lipid was 1:1 (mg/mg). For in vivo imaging studies, the nanoparticle contrast agent was administered intravenously via the tail vein at an iodine dose of 2.2 mg/gm body weight (2.2 mg total lipid/kg), delivered over a 1–2 minute period.

### Micro-CT

**Fig 1:**
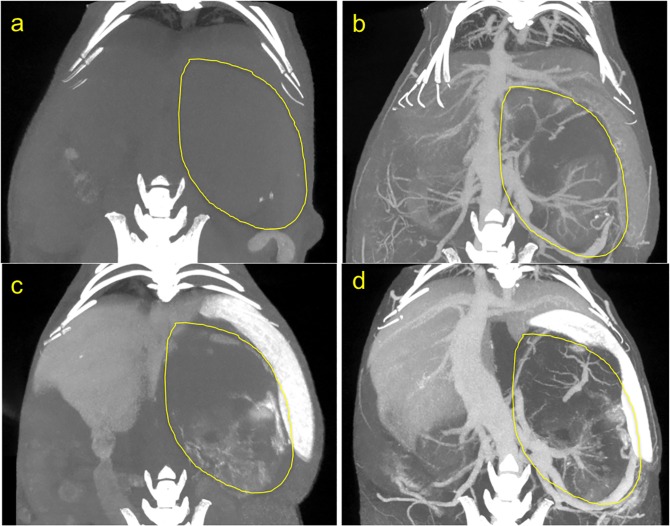
CT scan images of orthotopic tumor in mouse abdomen. Yellow line outlines the tumor. (a) Pre contrast injection. (b) immediately post contrast injection, showing the abdominal vasculature including vessels within the tumor. (c) 5 days post contrast injection, showing extravasated contrast within the tumor margin, and highlighting the liver and spleen, organs of the reticulo-endothelial system (RES) involved in clearance of the contrast agent. (d) After a 2^nd^ dose of contrast, thus depicting both the vasculature and the intratumoral extravasate.

Imaging was performed on a small animal micro-CT system (Inveon, Siemens Inc., Knoxville, TN, USA). The animals were placed prone on the scanner bed and scanned while free breathing under anesthesia using 1.5–2.5% isoflurane delivered by face-cone. An electrical resistive heating element was used to maintain and control body temperature during the entire imaging session. The respiratory rate was monitored using a pressure-pad placed under the animal in the abdominal region. CT image acquisition was performed using the following parameters set: 70 kVp peak voltage, 0.5 mA tube current, 850 ms X-ray exposure time, 540 projections over a 360 degree rotation, resulting in a 20 minute scan time. The estimated radiation dose, determined using a point source dosimeter, was 1.4 Gy. Images were reconstructed by filtered back-projection, using the COBRA v6.1 software package (EXXIM Computing Corp., Livermore, CA, USA), at an isotropic resolution of 35 μm. 60 animals inoculated with tumor cells (NGP or SH-SY5Y) were divided into four groups. Group 1 animals (n = 6) were imaged at one week, group 2 (n = 6) at two weeks, group 3 (n = 12) at three weeks and group 4 (n = 6) at four weeks, post-tumor implantation. Fig 1 shows a typical set of scans, before contrast injection, immediately after contrast injection showing the vasculature, 5 days post contrast injection showing the extravasated contrast and highlighting the leak within the tumor, and finally post a second dose of contrast showing the vasculature and the extravasated contrast in the same field. Phantoms containing solutions of known iodine concentration were placed in the field of view of every scan, enabling quantification of the iodine uptake in tumors. Each animal underwent a pre-contrast scan followed by administration of the contrast agent. An immediate post-contrast scan was performed within one hour after administration of the contrast agent (acute post-contrast). Thereafter, the animals were scanned four days (for SH-SY5Y tumor-bearing animals) or five days (for NGP tumor-bearing animals) post-administration of the contrast agent (delayed post-contrast). The animals were subsequently sacrificed by CO_2_ inhalation followed by cervical dislocation, and both the kidneys (tumor-implanted and contralateral) were extracted and weighed. The kidneys were formalin-fixed, paraffin-embedded, sectioned and stained with hematoxylin and eosin. A board-certified pathologist reviewed the slides for the presence or absence of tumor.

### Data Analysis

Tumor weight was estimated by subtracting the weight of contralateral kidney from the weight of tumor-implanted kidney. Image segmentation was performed in Osirix (version 5.8.5 64-bit; Pixmeo, Bernex, Switzerland). Quantitative image analysis and statistical analysis was performed in MATLAB (version 7.13; Natick, MA, USA).

#### Determination of tumor volume

Tumor volumes were determined from CT images. The tumor-implanted kidney and the contralateral kidney were manually segmented on CT images. Since the tumor margins within the kidney were not clearly visible on CT images, tumor volume was estimated by subtracting the segmented contralateral kidney volume from the segmented tumor-implanted kidney volume, *V*_*tumor*_ = *V*_*tumor*+*kidney*_ − *V*_*contralateral kidney*_.

#### Determination of CT signal enhancement

Blood CT signal, measured in Hounsfield Units (HU), was determined in the abdominal region of the inferior vena cava. For quantitative analysis of CT images, CT signal was converted into iodine concentration using a normalization factor as per methods described previously [15,16]. The normalization factor was determined by the slope of the line that best fit the plot of CT signal and iodine concentration in the phantom series. The signal enhancement in each tumor-implanted kidney was used to determine the intratumoral nanoparticle distribution and calculate total liposomal-iodine levels in the tumor.

#### Determination of leak volume

**Fig 2:**
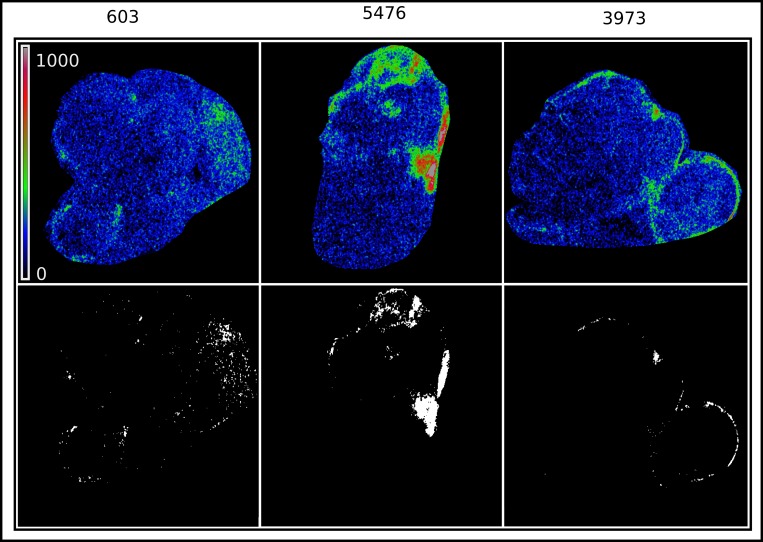
Maximum intensity projections of three segmented tumors, of similar tumor weight and size (~ 1.2 g, ~1cm major axis), showing iodine deposition in the tumors as a color map (top row). Thresholding the iodine signal at 3σ renders the locations of high iodine deposition (white) and the rest of the tumor (dark). The high iodine deposition locations are designated as extravascular leak.

The separation of tumor margins from healthy kidney margins in the delayed post-contrast CT images was not possible in both the orthotopic tumor models because the kidney was essentially obliterated by the tumor. As a result, 3D analysis of nanoparticle uptake was performed on the entire tumor-bearing kidney in those mice where tumor weights were greater than contralateral kidney weight (15 mice in NGP group and 4 mice in SH-SY5Y group). The intensity distribution of CT signal in tumor-implanted kidney and contralateral kidney was well-represented by a Gaussian (normal) distribution. Each image was thresholded using a level 3σ above the mean CT signal, thereby differentiating contrast-enhanced pixels from background pixels. An example of such thresholding, and the identification of selected pixels as representing vascular leak is shown in Fig 2. The volume of leak *V*_*leak*_ is calculated by integrating the volume of voxels contained in the sub-volume above the threshold.

#### 3D analysis of intratumoral nanoparticle distribution

**Fig 3:**
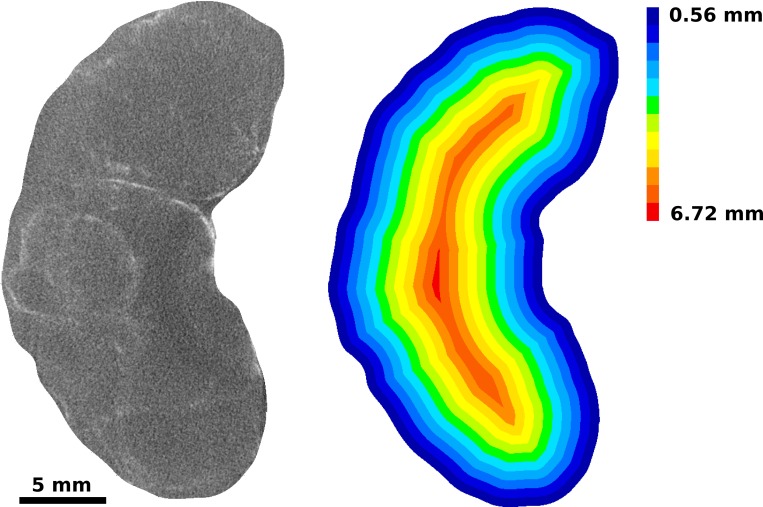
Radial discretization of tumor volume into 140μm thick concentric sections. The process begins at the periphery of the tumor and creating a sub-volume that penetrates 140μm into the interior of the tumor. The process is repeated for the remaining portion of the tumor until the remaining portion has a dimension less than 140μm.

A concentric shell model was used to determine the spatial 3D distribution of liposomal-iodine contrast agent within the tumor. The tumor-implanted kidney was divided into multiple concentric shells (140 μm thickness = 4x the voxel size), beginning with an outer shell that matches the outer surface of the segmented volume, and ending when the remaining volume inside the shell had any one dimension smaller than the shell thickness of 140 μm. The number of shells times the shell thickness was used as an estimate of the minor radius *r*_*minor*_ of the tumor. Surface area of the tumor was estimated as $A_{tumor}=\frac{V_{tumor}}{r_{minor}}$. An example is shown in Fig 3. Iodine intensity in each shell is calculated by summing the iodine signal within each shell. Similarly, the volume of leak in each shell is calculated by summing the volume of thresholded voxels within the shell. Cumulative iodine amounts within a shell radius is calculated by summing all iodine amounts for the chosen, and smaller shells. Shell radii are normalized to the largest radius, thus permitting comparison of the distributions within tumors of different sizes. R30 and R50, normalized radii within which 30% and 50% of the cumulative iodine was deposited, were estimated from the cumulative distributions. The extent of variability in uptake as a function of each of these parameters was estimated by fitting the data to generalized higher degree (n ≤ 5) polynomials and selecting the fit with the best correlation coefficient. These fits are not meant to depict the actual relationship between abcissa and ordinate, but are rather, a guide for the eye, and the scatter of the data around the line an indication of the variability of the uptake with respect to the ordinate.

## Results and Discussion

In the NGP group, 4/6 mice showed tumors at day 19, 9/12 mice showed tumors at day 26 and 6/6 mice showed tumors at day 33 post-implantation of tumors cells. In the SH-SY5Y group, 1/6 mice showed tumors at day 12, 4/6 mice showed tumors at day 19, 9/12 mice showed tumors at day 26 and 3/6 mice showed tumors at day 33 post-implantation of tumor cells. NGP tumors demonstrated rapid tumor growth compared to SH-SY5Y tumors. By day 26, the NGP tumors had grown to approximately five times the size of the normal (contralateral) kidney (0.21±0.04 g). 17/19 of histologically visible tumors in the NGP group and 8/17 of histologically visible tumors in the SH-SY5Y group showed signal enhancement in delayed post-contrast CT images.

**Fig 4:**
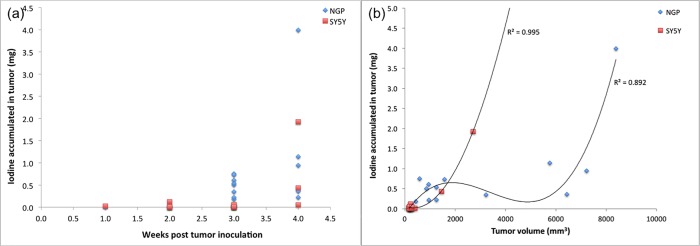
(a) Iodine uptake in whole tumors (mg), measured by integrating the CT intensity within the tumor volume, and applying the normalization factor. Iodine uptake is low and consistent in both tumor lines (NGP and SH-SY5Y) till 2 weeks post inoculation. At 3 weeks post inoculation, significant variability in uptake is seen in the NGP tumors, while it remains low and constant in the slower growing SH-SY5Y tumors. By week 4, both tumor types show significant variability. (b) Iodine uptake in whole tumors (mg) as a function of tumor volume estimated from CT image. Lines are the best fit polynomial to the data, as a guide to the eye. The scatter of the NGP data around the line suggests that tumor volume is not predictive of the uptake in NGP tumors, while the high degree of correlation of the SH-SY5Y data with the line suggests that tumor volume is predictive of uptake for the relatively slow growing SH-SY5Y tumors.

Total tumor uptake for both SH-SY5Y and NGP tumors, as a function of tumor age post inoculation is shown in Fig 4A, NGP tumors demonstrate highly variable uptake after 2 weeks post inoculation, while the slower growing SH-SY5Y tumors demonstrate relatively low and constant uptake through 3 weeks post inoculation, and begin to demonstrate uptake variability at the 4 week point.

Fig 4B shows the dependence of iodine uptake as a function of tumor volume in both tumor types. The uptake in SH-SY5Y tumors, however, appears to be dependent on tumor volume *V*_*tumor*_, suggesting a uniform distribution of vascular leak throughout the tumor. However, the more rapidly growing NGP tumors do not exhibit this behavior, and uptake appears to be independent of the tumor volume. While the plots are presented in terms of the nanoparticle payload i.e. mg of iodine, these also provide a direct quantitative measure of nanoparticle uptake due to equivalent iodine to total lipid ratio (on a weight basis) in the nanoparticle contrast agent.

**Fig 5:**
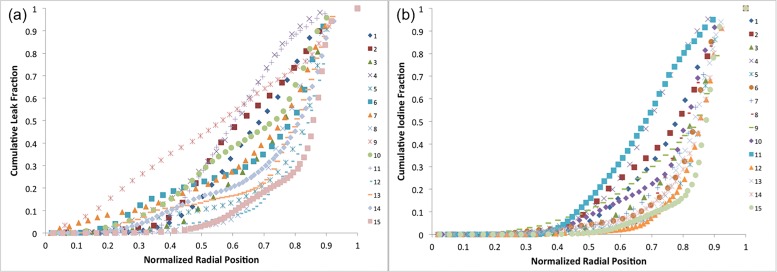
(a) Normalized cumulative leak volume as a function of normalized radial position for 15 individual NGP tumors ranging in age from 2 to 4 weeks post inoculation, and ranging in volume from 150 to 8500 mm3. (b) Normalized cumulative iodine uptake as a function of normalized radial position for 15 individual NGP tumors ranging in age from 2 to 4 weeks post inoculation, and ranging in volume from 150 to 8500mm3.

**Fig 6:**
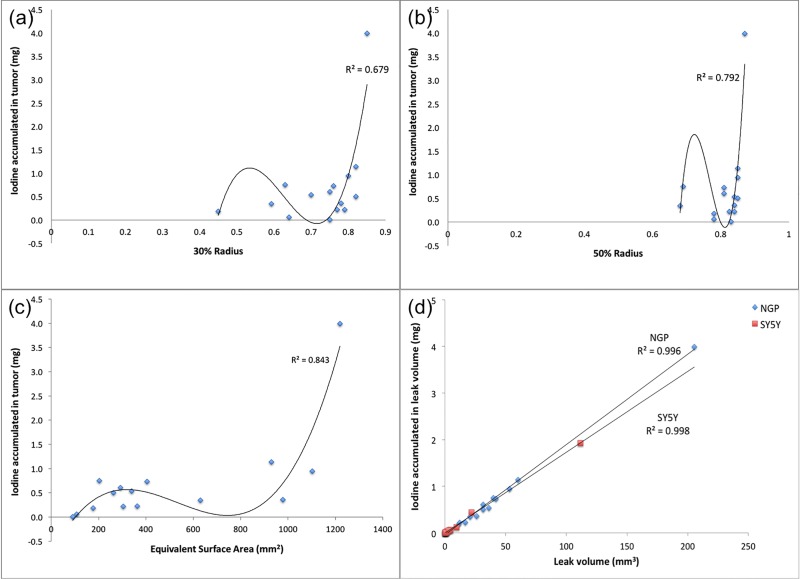
(a) Iodine uptake in NGP tumors as a function of R30, the radius within which 30% of the total uptake is contained. Line is the best fit polynomial to the data, and the scatter around the line suggests that R30 is not predictive of the uptake. (b) Iodine uptake in NGP tumors as a function of R50, the radius within which 50% of the total uptake is contained. Line is the best fit polynomial to the data, and the scatter around the line suggests that R50 is not predictive of the uptake. (c) Iodine uptake in NGP tumors as a function of A_tumor_, the estimated surface area of the tumor. Line is the best fit polynomial to the data, and the scatter around the line suggests that tumor surface area is not predictive of the uptake. (d) Uptake of Iodine payload in NGP and SH-SY5Y tumors as a function of the leak volume as measured from the CT image. Straight lines are least-squares best fits to the data. The high correlation coefficient suggests that the leak volume is predictive of the uptake. Further, the slope of the lines correlates well with the anticipated concentration of the agent within the leak zone.

The radial distribution of leak volume and uptake in fifteen individual NGP tumors are shown in Fig 5A and 5B. The data are plotted as normalized cumulative leak volume and uptake as a function of normalized radius. Both leak volume and iodine uptake show dramatic variability in their patterns of distribution. #15, for example, exhibits the majority of liposomal-iodine uptake in the outermost 20% of the radius, while #11 and #4 have close to 50% of the liposomal-iodine uptake in the inner 60% of the radius. This suggested that the overall liposomal-iodine uptake could be decomposed into surface uptake (at the periphery of the tumor) and inner, or core, uptake, within the bulk of the tumor. We therefore calculated R30 and R50 (normalized radii within 30% and 50% respectively, of the total iodine uptake was contained) as measures of the core uptake in each tumor. However, neither of these parameters, nor the tumor area *A*_*tumor*_, appears to be predictive of the uptake as evident by the poor correlation coefficient values (R^2^) (Fig 6A, 6B and 6C), suggesting that neither the surface area nor the core uptake determines the distribution of nanoparticle uptake in the tumor.

Since none of the tested tumor geometric parameters appear to be predictive of uptake in NGP tumors, we resort to a direct measure of the leak volume, *V*_*leak*_, as measured by nanoparticle contrast extravasation. Fig 6D shows that for both NGP and SH-SY5Y, the uptake is directly proportional to the leak volume. Straight lines in Fig 6D are least-squares fits to the data. The slopes of these lines are a measure of the average concentration (mg/mm^3^) in the leaks. The leak of Stealth nanoparticles into tumors is attributed to passive extravasation, the well-known “Enhanced Permeation and Retention Effect” or EPR. One anticipates therefore that tumor uptake of nanoparticles and nanoparticle payload should be a function of the permeable surface area of the intratumoral vasculature. The concentration of tracer in the tumor extravascular space should be given by a balance between the extravasation rate and clearance from the extravascular space. Assuming that in the duration of this experiment (~96 hours), clearance of extravasated particles from the extravascular space is small, the concentration should be given by the average blood pool concentration of the nanoparticle agent, adjusted for volume change of the extravasate due to lymphatic drainage. Assuming a blood half-life of 48 hours (or a first order decay constant of 0.014hr^-1^), the average concentration in the blood pool over 96 hours is given by $\frac{\int_{0}^{96}Cdt}{96}=\frac{\int_{0}^{96}C_{0}e^{-kt}dt}{96}$ = 14.9 mg Iodine/ml, which is 20–25% lower than the estimated values from Fig 6D of 17.3 mg Iodine/ml for SH-SY5Y tumors and 19.4 mg Iodine/ml for NGP tumors. If the extravasate were to lose 20–25% of its volume to lymphatic drainage over the duration of the experiment, this would be consistent with the estimated concentrations.

The results of this study are consistent with our previous work [6–9]. In rapidly growing NGP tumors, there is a high degree of variability of liposomal nanoparticle uptake by the tumors. One anticipates that this variability would translate into an equivalent variability of uptake of nanoparticle borne drug, resulting in highly variable therapeutic response. In slow growing SH-SY5Y tumors, there is a lower degree of variability, and further, the variable uptake is well predicted by a simple geometric parameter, the tumor volume. In rapidly growing NGP tumors however, no such simple geometric parameter that is predictive of nanoparticle uptake is found. However, the leak volume, or the volume of tumor tissue that is reached by the extravasate, appears to be directly predictive of the total uptake. The leak volume is a function of tumor’s vascular fraction and vessel permeability. As seen with the rapidly growing NGP tumors, a large tumor volume does not necessarily reflect a higher accumulation of nanoparticle probe or high leak volume. The data are also consistent with the current model of the EPR effect, whereby the intratumoral levels of payload are determined by the balance between extravasation rate and clearance from the extravascular space. Further, this relationship appears to be valid for the slow growing SH-SY5Y tumors as well. For both tumor types, a simple calculation based on reasonable assumptions about the pharmacokinetics of Stealth liposomes leads to a quantitative prediction of the concentration of payload in the extravasate. As demonstrated in this work, the determination of the leak volume, or for that matter the actual intratumoral deposition or uptake of the nanoparticle payload itself, is relatively easy to do using a nanoparticle imaging agent such as the one described in this work, and the uptake prediction is accurate regardless of the growth rate of the tumor. Studies are currently ongoing to test the utility of this imaging methodology for predicting the uptake of PEGylated liposomal doxorubicin in mouse models of neuroblastoma.

## Conclusion

The current study investigated the uptake and intratumoral distribution of liposomal nanoparticles in orthotopic xenograft mouse models of pediatric neuroblastoma. Similar to the models of adult solid tumors, we demonstrated, using CT imaging and a liposomal contrast agent, that pediatric solid tumors exhibit heterogeneous uptake and intratumoral distribution of nanoparticles. Furthermore, using high-resolution CT imaging, we were able to visualize and quantify the uptake and intratumoral distribution of nanoparticles and show that leak volume is directly predictive of total nanoparticle uptake in slow growing and fast growing neuroblastoma models.
